# Study on the Stability of DeoxyArbutin in an Anhydrous Emulsion System

**DOI:** 10.3390/ijms12095946

**Published:** 2011-09-15

**Authors:** Chih-Chien Lin, Chao-Hsun Yang, Nai-Fang Chang, Pey-Shiuan Wu, Yi-Shyan Chen, Shu-Mei Lee, Chiu-Wen Chen

**Affiliations:** 1 Department of Cosmetic Science, Providence University, 200 Chung-Chi Road, Shalu, Taichung 43301, Taiwan; E-Mails: chyang@pu.edu.tw (C.-H.Y.); nfchang@pu.edu.tw (N.-F.C.); jwu2@pu.edu.tw (P.-S.W.); yishyan@gm.pu.edu.tw (Y.-S.C.); rita770115@hotmail.com (C.-W.C.); 2 Department of Cosmetic Science and Management, Mackay Medicine, Nursing and Management College, 92 Shengjing Road, Beitou, Taipei 11260, Taiwan; E-Mail: s107@eip.mkc.edu.tw

**Keywords:** anhydrous emulsion system, deoxyArbutin, hydroquinone, skin whitening, stability

## Abstract

The skin-whitening agent, deoxyArbutin, is a potent tyrosinase inhibitor that is safer than hydroquinone and arbutin. However, it is thermolabile in aqueous solutions, where it decomposes to hydroquinone. Pharmaceutical and cosmetic emulsions are normally oil-in-water (o/w) or water-in-oil (w/o) systems; however, emulsions can be formulated with no aqueous phase to produce an anhydrous emulsion system. An anhydrous emulsion system could offer a stable vehicle for compounds that are sensitive to hydrolysis or oxidation. Therefore, to enhance the stability of deoxyArbutin in formulations, we chose the polyol-in-silicone, anhydrous emulsion system as the basic formulation for investigation. The quantity of deoxyArbutin and the accumulation of hydroquinone in both hydrous and anhydrous emulsions at various temperatures were analyzed through an established high performance liquid chromatographic (HPLC) method. The results indicated that water increased the decomposition of deoxyArbutin in the formulations and that the polyol-in-silicone, oil-based, anhydrous emulsion system provided a relatively stable surrounding for the deoxyArbutin that delayed its degradation at 25 °C and 45 °C. Moreover, the composition of the inner hydrophilic phase, containing different amounts of glycerin and propylene glycol, affected the stability of deoxyArbutin. Thus, these results will be beneficial when using deoxyArbutin in cosmetics and medicines in the future.

## 1. Introduction

**Figure 1:**
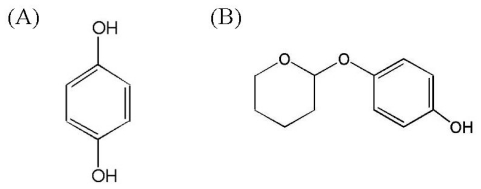
Chemical structure of hydroquinone (**A**) and deoxyArbutin (**B**).

Tyrosinase is an enzyme with a copper center that is widely expressed in many life forms and is mainly involved in the formation of pigments, such as melanin and other polyphenolic compounds. The biosynthesis of melanin in melanocytes might be affected by cellular tyrosinase activity [1,2]. In addition, increased production and accumulation of melanin leads to many hyperpigmentation diseases such as melasma, solar lentigines, and post-inflammatory hyperpigmentation. Therefore, investigation into depigmentation mechanisms and the clinical aspects of skin whitening agents is very important [3,4]. Various compounds have demonstrated inhibitory effects on melanogenesis through inhibition of the enzymatic activity of tyrosinase [5–7]. For example, hydroquinone (Figure 1A) and deoxyArbutin (dA; Figure 1B) are both potent skin-whitening agents that are capable of suppressing the function of tyrosinase. DeoxyArbutin (4-[(tetrahydro-2H-pyran-2-yl)oxy]phenol) was first shown by Boissy *et al.* to exhibit greater inhibition of tyrosinase activity and to be safer than hydroquinone and arbutin [8]. Moreover, deoxyArbutin demonstrated fast and persistent skin lightening effects both in an animal model and in a human trial [9,10]. However, in our previous study, we found that this skin whitening agent was thermolabile in aqueous solutions and decomposes to hydroquinone under these conditions [11]. Instability in water posed developmental and practical problems for using deoxyArbutin in cosmetics and medication. Thus, enhancing the stability of this skin-whitening agent is important for its development.

**Figure 2:**
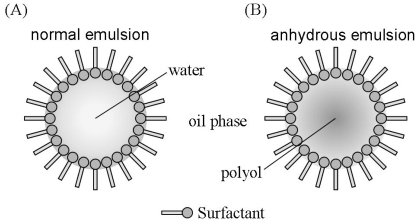
Illustration of a normal emulsion (**A**) and an anhydrous emulsion (**B**).

Pharmaceutical and cosmetic emulsions are normally oil-in-water (o/w) or water-in-oil (w/o) systems (Figure 2A). However, emulsions can be formulated with no aqueous phase to produce an anhydrous, non-aqueous, oil-in-oil or oil-polar solvent emulsion system [12]. A phospholipid-based [13], petroleum-formamide/dimethyl formamide/dimethyl sulfoxide-based [14], hydrocarbon-formamidebased [15] or silicone-based [12] system could offer a stable vehicle for transporting active compounds that are sensitive to hydrolysis or oxidation. For instance, a polyol-in-silicone anhydrous emulsion system comprised of a polar phase with polyol, which replaces water, and a nonpolar phase with silicone oil (Figure 2B) could be used in this manner. Specifically, the polyol in similar systems has regularly been selected as propylene glycol, butylene glycol or glycerin.

To enhance the stability of deoxyArbutin in formulations, we chose the polyol-in-silicone anhydrous emulsion system as the basic formulation for investigation. The quantity of deoxyArbutin in both a normal (hydrous) emulsion and an anhydrous emulsion at various temperatures were analyzed through an established high performance liquid chromatographic (HPLC) method. Moreover, the accumulation of hydroquinone in these formulations were analyzed and compared to each other.

## 2. Results and Discussion

The major objective of this work was to examine the stability of deoxyArbutin in an anhydrous emulsion system. Therefore, in the first part of the study, we used two polyol-in-silicone anhydrous emulsions in formulations containing deoxyArbutin. Secondly, we used an established HPLC method to confirm the quantity of deoxyArbutin and hydroquinone in these formulations at various temperatures. Thus, the difference in the stability of deoxyArbutin in these normal and anhydrous emulsions will be revealed by this study.

### 2.1. Preparation of DeoxyArbutin-Containing Formulations

The compositions of the anhydrous (anH-1/2) and hydrous (H-1/2) formulations used in this study are listed in Table 1. We used 4% or 2% cetyl dimethicone copolyol as the emulsifier to formulate the silicone-based formulations 1 (anH-1/H-1) and 2 (anH-2/H-2), respectively. The outer phase, or oil phase, was produced by adding cyclomethicone with or without stearyl dimethicone and isostearyl isostearate. Additionally, the inner phase, or hydrophilic phase, was composed of deoxyArbutin (3%), propylene glycol and glycerin, with or without water. In this phase, propylene glycol and glycerin provide the solubility of deoxyArbutin. In addition, anhydrous (anH-1/2) and hydrous (H-1/2) formulations were defined according to the water content of hydrophilic phase in formulations. Thus, we prepared two polyol-in-oil anhydrous emulsions and two water-in-oil (w/o) hydrous emulsions for this study (Table 1).

The prepared formulations had no obvious difference in appearance between each other. All prepared formulations were centrifuged at 2330× g for 20 min to check the stability of the formulations. The results indicated that the formulations made in this study were stable (no phase separation occurs) and remained stable until the experiment was completed (14 weeks) and after the formulations were incubated at 45 °C (data not shown).

### 2.2. Stability of DeoxyArbutin in Formulations at Low Temperature

**Figure 3:**
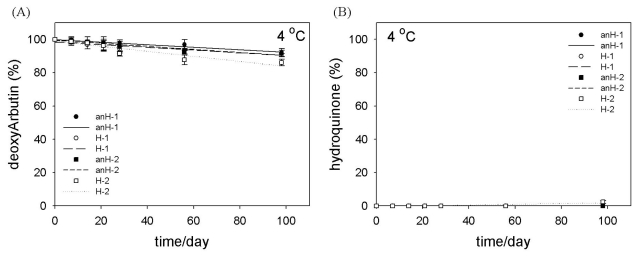
Stability of deoxyArbutin (**A**) and the accumulation of hydroquinone (**B**) at 4 °C (low temperature) in various formulations.

To verify the stability of deoxyArbutin in an anhydrous emulsion system, we placed the prepared formulations at various temperatures and then analyzed the amount of deoxyArbutin and hydroquinone in each formulation on each of the selected days for a total of 98 days (14 weeks). In all of the formulations, concentrations of deoxyArbutin were slightly decreased during the time course at 4 °C, and the lowest amount of deoxyArbutin, in the H-2 formulation on the 98th day, was 86.08 ± 1.97% (Figure 3A). Notably, hydroquinone was not detectable (N.D.) in any of the formulations at any time point at 4 °C, except for the H-2 formulation on the 98th day (2.41 ± 0.84%; Figure 3B).

Although the difference in the stability of deoxyArbutin in the normal and anhydrous emulsions was not evident at 4 °C, there was a slight increase in the stability of deoxyArbutin when the formulation was prepared without water (Figure 3A). However, this result also indicated that formulated deoxyArbutin kept at a low temperature underwent degradation.

### 2.3. Stability of DeoxyArbutin in Formulations at Moderate Temperature

**Figure 4:**
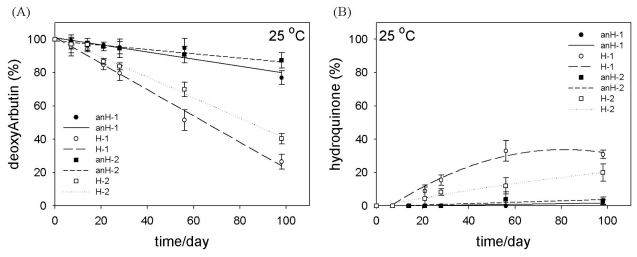
Stability of deoxyArbutin (**A**) and the accumulation of hydroquinone (**B**) at 25 °C (moderate temperature) in various formulations.

At 25 °C, the amount of deoxyArbutin in both anhydrous formulations (anH-1/2) was higher than that in the hydrous formulations (H-1/2; Figure 4A). On the last day of this experiment, the retention percentages of deoxyArbutin in the anH-1 and anH-2 formulations were 76.86 ± 4.11% and 87.29 ± 4.63%, respectively. Comparatively, the retention percentage of deoxyArbutin in the H-1 and H-2 formulations on the 98th day were only 26.49 ± 4.51% and 40.28 ± 3.07%, respectively; thus, we concluded that deoxyArbutin in an anhydrous emulsion system was more stable. In addition, the accumulation of hydroquinone in these formulations was inversely correlated with the decay of deoxyArbutin, such as in formulation H-1, which had the lowest retention percentage of deoxyArbutin and the most abundant hydroquinone accumulation (Figure 4A and 4B). This decomposition process in aqueous solutions had been demonstrated in our previous study [11].

Moreover, deoxyArbutin was more stable in a water or polyol-in-silicone oil emulsion at 25 °C (higher than 84.62% in all formulations on the 21st day) than what was seen in our previous study where deoxyArbutin had been dissolved in water with 10% propylene glycol (49.42%) [11]. This result indicated that the droplet structures of emulsions provided a stable environment for deoxyArbutin and that a similar phenomenon of enhanced drug stability was revealed in numerous emulsion systems [16–18].

### 2.4. Stability of DeoxyArbutin in Formulations at High Temperature

**Figure 5:**
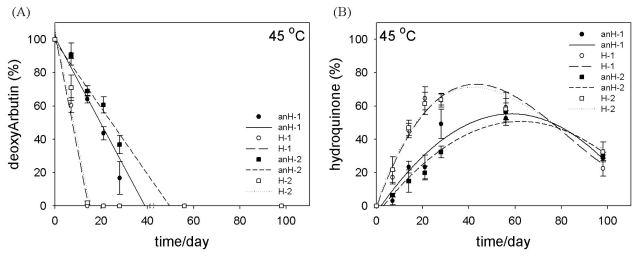
Stability of deoxyArbutin (**A**) and the accumulation of hydroquinone (**B**) at 45 °C (high temperature) in various formulations.

At 45 °C and in all formulations, deoxyArbutin decomposed quickly (Figure 5A); however, the most unstable surroundings were the water-containing formulations (H-1/2). Moreover, deoxyArbutin was completely decayed within two weeks in the H-1 and H-2 formulations at 45 °C, but deoxyArbutin in the anH-1 and anH-2 formulations persisted for approximately 40 to 50 days (Figure 5A). These results demonstrated that deoxyArbutin in an anhydrous emulsion system was more stable than in a hydrous emulsion system. Furthermore, hydroquinone accumulated in all formulations when maintained at this temperature (Figure 5B). Although the percentage of hydroquinone was decreased in the high temperature environment at the end of this experiment, the relationship between deoxyArbutin decomposition and hydroquinone accumulation was still evident (Figure 5A and 5B). In addition, hydroquinone at high temperature may also undergo some decomposition process. Thus, the content of hydroquinone was decreased after a period of 40–60 days at 45 °C (Figure 5B).

Furthermore, when comparing formulations 1 and 2, the retention percentage of deoxyArbutin in H-2 was higher than in H-1 at 25 °C (Figure 4A), and the retention percentage of deoxyArbutin in anH-2 was higher than that in anH-1 at 45 °C (Figure 5A). This consequence possibly resulted from the diverse compositions of formulations 1 and 2. For instance, formulation 1 had a higher amount of glycerin and a lower amount of propylene glycol (Table 1). This difference provided diverse hydroxyl groups in the inner hydrophilic phase of these two types of formulations. Furthermore, the difference in the composition of the oil phase could have also presented a minor effect on the stability of deoxyArbutin in these formulations (Table 1).

In summary, water may increase the decomposition of deoxyArbutin in formulations; thus, the anhydrous emulsion system could provide a relatively stable surrounding that could delay degradation of deoxyArbutin at temperatures of 25 and 45 °C. The results from this study are important for improving the formulation for deoxyArbutin. Besides, many factors such as pH value or other ingredients in the emulsions also affect the stability of deoxyArbutin. Moreover, the adding of stabilizer or antioxidant into the formulation or with oil-in-oil formulations may further enhance the stability of deoxyArbutin. In addition, these strategies can also apply to other compounds that are sensitive to hydrolysis. We are currently investigating these potential methods to improve the applications of deoxyArbutin.

## 3. Experimental Section

### 3.1. Materials

DeoxyArbutin (99.9%) was purchased from Denjelly Co., Ltd (Miaoli, Taiwan, R.O.C), and hydroquinone (99.8%) was purchased from Wako Pure Chemical Industries (Osaka, Japan). HPLC-grade methanol was purchased from Merck (Darmstadt, Germany), cetyl dimethicone copolyol and stearyl dimethicone were purchased from EVONIK Goldschmidt GmbH (Essen, Germany), cyclomethicone was purchased from Dow Corning Corporation (Midland, MI, USA), and isostearyl isostearate was purchased from Corum Inc. (Taipei, Taiwan, R.O.C). Propylene glycol, glycerin and other chemicals were purchased from Sigma-Aldrich (St. Louis, MO, USA), and deionized distilled water (ddH_2_O), which was used for solutions, was obtained with a Milli-Q system (Millipore, Bedford, MA, USA).

### 3.2. Preparation of Formulations and Emulsion Stability Test

The compositions of the formulations, including the hydrous and anhydrous emulsions, are listed in Table 1. DeoxyArbutin (3%) was first pre-dissolved in propylene glycol, and all formulations were prepared by premixing the hydrophilic and hydrophobic phases. After premixing, the hydrophilic and hydrophobic components were emulsified via a high-shear mixer at an agitation speed of 200 rpm for 2 min. Subsequently, increase the agitation speed at 1000 rpm for 5 min to complete the emulsification. To test the stability of emulsions, all prepared formulations were centrifuged at 2,330× g for 20 min to check their stability. Besides, at every time point, the formulations were also determined whether the phase separation occurs to confirm the emulsions are stable.

### 3.3. Sample Preparation and High Performance Liquid Chromatography (HPLC) Analysis

For sample preparation, 0.25 g of emulsion was dissolved in 50 mL of methanol and then sonicated for 30 min in a water bath sonicator. The extracted sample was subsequently diluted (1:24 (v/v)) for analysis in pure methanol. The analytic procedures for deoxyArbutin and hydroquinone were performed according to our previous study [11]. Specifically, each 20-μL sample was injected into the HPLC apparatus (Agilent 1100 series, USA), which utilized a pre-packed C18 reversed-phase column (Mightysil RP-18, GP 250-4.6, Kanto Chemical, Tokyo, Japan). The mobile phase, composed of methanol and water (60:40 (v/v), pH 7), was run at a flow rate of 1 mL/min The solvents were filtered separately through a 0.45 mm filter (Millipore) and mixed in the desired proportions. The UV wavelength was set to 280 nm for detection of both deoxyArbutin and hydroquinone, and the ratios of the compound peak areas to the internal standard peak areas were calculated using the corresponding concentrations. These ratios were then used to obtain the calibration graph. In the determination of hydroquinone, the percentage of hydroquinone was calculated by the molar ratio of deoxyArbutin to hydroquinone, *i.e*., 3 g of deoxyArbutin can completely decompose to 1.701 g of hydroquinone.

### 3.4. Stability Studies of DeoxyArbutin in the Emulsion System

The normal and anhydrous formulations containing 3% deoxyArbutin were placed in glass bottles and kept in the dark. Once in an incubator, these samples were exposed to the following temperatures: 4 °C (low temperature), 25 °C (moderate temperature) and 45 °C (high temperature). At each of the designated time points, the formulations were analyzed using the previously described HPLC method to monitor the stability of deoxyArbutin and hydroquinone in these emulsion systems.

### 3.5. Statistical Analysis

All data were obtained in three independent experiments and then analyzed to determine the mean values. Statistical comparisons of the means and simple correlation coefficients were performed using the Student’s *t*-test.

## 4. Conclusions

In conclusion, we have demonstrated that water may enhance the decomposition of deoxyArbutin in formulations and that a polyol-in-silicone oil-based anhydrous emulsion system can provide a relatively stable surrounding that delays the degradation of deoxyArbutin at temperatures of 25 and 45 °C. Moreover, the composition of the inner hydrophilic phase, which contains a different amount of glycerin and propylene glycol, might affect the stability of deoxyArbutin. Therefore, the results from this study can help in determining practical uses for deoxyArbutin in cosmetics and medicines in the future.

## Figures and Tables

**Table 1:** Compositions of formulations used in this study.

formulation	**ingredients**							
	dA	cetyl dimethicone copolyol	cyclomethicone	stearyl dimethicone	isostearyl isostearate	propylene glycol	glycerin	deionized water
anH-1	3%	4%	17%	0%	0%	46%	30%	0%
H-1	3%	4%	17%	0%	0%	46%	10%	20%
anH-2	3%	2%	14%	3%	2%	70%	6%	0%
H-2	3%	2%	14%	3%	2%	50%	6%	20%

anH: anhydrous emulsion, H: hydrous emulsion.
